# P01 Cluster randomized controlled trial (RCT) of Air Filtration to prevent Respiratory Infections (including COVID-19) in care homes: the AFRI-c trial

**DOI:** 10.1093/jacamr/dlae217.005

**Published:** 2025-01-23

**Authors:** Alastair Hay, Rachel Brierley, Nick Turner, Joanna Thorn, Sophie Rees, Jodi Taylor, Tomas Welsh, Emily Henderson, Jane Sprackman, Gemma Morgan, Craig Riddington, Laurel Campbell-Smith, Claire Brown, Derren Ready, Graziella Mazza, Eleanor Gidman, Ruth Kipping

**Affiliations:** University of Bristol, Bristol, UK; University of Bristol, Bristol, UK; University of Bristol, Bristol, UK; University of Bristol, Bristol, UK; University of Bristol, Bristol, UK; University of Bristol, Bristol, UK; University of Bristol, Bristol, UK; University of Bristol, Bristol, UK; University of Bristol, Bristol, UK; University of Bristol, Bristol, UK; University of Bristol, Bristol, UK; University of Bristol, Bristol, UK; University of Bristol, Bristol, UK; University of Bristol, Bristol, UK; University of Bristol, Bristol, UK; University of Bristol, Bristol, UK; University of Bristol, Bristol, UK

## Abstract

**Background:**

Respiratory tract infections (RTIs) are common in people in care homes. Transmission can be through direct contact or airborne particles. Portable high-efficiency-particulate-air (HEPA) filtration units remove airborne microbial particles, but it is unclear if this is sufficient to reduce infections.

**Objectives:**

To investigate the effectiveness of portable HEPA filtration units to reduce RTIs in care home residents.

**Methods:**

Ninety residential care homes were randomized to HEPA filtration units in communal rooms and private bedrooms or usual care. Primary outcome: number of RTIs recorded by care home staff. Secondary outcomes: other infections; falls/near falls; laboratory confirmed infections; hospitalizations; and staff sickness.

**Results:**

A total of 43 care homes were randomized to intervention, 47 to usual care. Overall, 43% and 45% of intervention and control group care homes provided nursing care; 86% and 87% dementia care; and median (IQR) number of communal rooms were 4 (3–6) and 4 (3–5). A total of 1158 residents were consented, of whom 34 (3%) moved away; 172 (15%) died; and 5 stopped participating. Primary outcome data is 98% complete with 195 463 ‘resident days’ symptom data collected. Comparing intervention and control group residents: median (IQR) age was 87 (81–92) and 88 (82–92) years; 70% and 71% were female; 35% and 43% were receiving nursing care; 58% and 57% had dementia; frailty scores were 6 (4–7) and 6 (5–7); 95% and 94% received influenza vaccines; and 97% and 96% received COVID vaccines.

**Conclusions:**

Full results will be available for conference. This global first RCT will provide invaluable evidence regarding the role of portable HEPA filtration to reduce RTIs in the care home setting.

